# Laser irradiation in water for the novel, scalable synthesis of black TiO*_x_* photocatalyst for environmental remediation

**DOI:** 10.3762/bjnano.8.21

**Published:** 2017-01-19

**Authors:** Massimo Zimbone, Giuseppe Cacciato, Mohamed Boutinguiza, Vittorio Privitera, Maria Grazia Grimaldi

**Affiliations:** 1 CNR-IMM, via S. Sofia 64, 95123 Catania, Italy; 2 Grupo de Aplicaciones Industriales de los Láseres, Departamento de Física Aplicada, E.T.S. Ingenieros Industriales de Vigo, Rúa Maxwell, s/n, Campus Universitario, 36310 Vigo, Spain; 3 Dipartimento di Fisica e Astronomia, Università di Catania, via S. Sofia 64, 95123 Catania, Italy

**Keywords:** black titania, laser irradiation in water, photocatalysis, TiO*_x_*, water treatment

## Abstract

Since 1970, TiO_2_ photocatalysis has been considered a possible alternative for sustainable water treatment. This is due to its material stability, abundance, nontoxicity and high activity. Unfortunately, its wide band gap (≈3.2 eV) in the UV portion of the spectrum makes it inefficient under solar illumination. Recently, so-called “black TiO_2_” has been proposed as a candidate to overcome this issue. However, typical synthesis routes require high hydrogen pressure and long annealing treatments. In this work, we present an industrially scalable synthesis of TiO_2_-based material based on laser irradiation. The resulting black TiO*_x_* shows a high activity and adsorbs visible radiation, overcoming the main concerns related to the use of TiO_2_ under solar irradiation. We employed a commercial high repetition rate green laser in order to synthesize a black TiO*_x_* layer and we demonstrate the scalability of the present methodology. The photocatalyst is composed of a nanostructured titanate film (TiO*_x_*) synthetized on a titanium foil, directly back-contacted to a layer of Pt nanoparticles (PtNps) deposited on the rear side of the same foil. The result is a monolithic photochemical diode with a stacked, layered structure (TiO*_x_*/Ti/PtNps). The resulting high photo-efficiency is ascribed to both the scavenging of electrons by Pt nanoparticles and the presence of trap surface states for holes in an amorphous hydrogenated TiO*_x_* layer.

## Introduction

The interest in titanium dioxide dates back in 1972, thanks to the pioneering work of Honda and Fujishima [1]. TiO_2_ has been widely used for both water splitting and mineralization of organic contaminants in solution [2]. The applications range from third generation solar cells [3] to material for air or water purification [4] to antifogging and self-cleaning surfaces [5–6]. The main advantage of this material is its high inertness (even in a corrosive environment), stability, abundance and nontoxicity. Nevertheless, its exploitation in environmental remediation, and in particular in water purification, is relatively limited due to its wide band gap (≈3.2 eV). This means that only 5% of the incoming solar radiation can be readily employed for water decontamination. In order to enhance the photocatalytic performance of TiO_2_ under visible (solar) irradiation, many efforts have been made in the last years, ranging from doping with N, C and transition metals [7–9], to coupling with narrow band gap semiconductor quantum dots [10], to the use of metal grafting [11–14] or plasmonic metal nanostructures [15–19] and the preparation of oxygen-deficient and/or hydrogen-rich TiO*_x_* [20–22]. We are interested, in particular, in this last approach.

Hydrogenated black TiO_2_ has attracted attention due to its low band gap and large optical absorption [20–21] in the visible and IR spectral range. This material has also encouraged theoretical studies due to its peculiarities related to the hydrogen insertion. In the case of TiO_2_ nanoparticles, hydrogen induces the amorphization of a layer at the surface and the creation of acceptor states. These states are responsible for the visibly dark coloration and, consequently, for sunlight absorption [23]. Unfortunately, this material is not suitable for industrial implementation since it requires up to 20 bar of hydrogen pressure and up to 15 days of annealing treatment, according to the most common synthesis techniques [21]. Recently, we proposed laser ablation in water as a synthesis route for efficient TiO_2_-based catalysts by using a high energy 1064 nm wavelength laser [22,24–25].

In the present work, we focus our attention on the synthesis of a nanostructured, black TiO*_x_* film on a Ti foil (TiO*_x_*/Ti) by using a high repetition rate green laser commonly used in industry and metallurgy. This laser has a shorter wavelength (532 nm vs 1064 nm) and a fluence (2 J/cm^2^ vs 20 J/cm^2^) and pulse energy (10 mJ/pulse vs 1 J/pulse) of an order of magnitude lower than our previously presented methodology [22]. We demonstrate the scalability of the reported methodology and open up the possible further exploitation of such a technology. In fact, several differences arose with respect to our previous work, mainly regarding the morphology and crystallinity of the obtained film. However, variations were not observed in the composition (stoichiometry) or the optical and catalytic properties of the material. In the present work, we are working near the threshold for the formation of the black layer of TiO*_x_*. This means that the laser supplies enough energy to induce melting and oxidation on the metallic titanium surface and, at the same time, the process is fast enough to allow the formation of substoichiometric oxides. We have demonstrated that the proposed methodology allows for the successful synthesis of a black TiO*_x_* material suitable for water purification applications. The morphological, structural and optical properties of the film surface were investigated. Moreover, we fabricated a photochemical diode with remarkable activity for the degradation of pollutants in water. The device is created by depositing a layer of Pt nanoparticles (PtNps) on the rear side of the TiO*_x_*/Ti substrate, producing a monolithic electrochemical cell [22]. The device consists of a stacked, layered structure: TiO*_x_*/Ti/PtNps. The photo-activity of the material is related to the trapping and scavenging of holes due to the amorphous black layer and to the scavenging of electrons caused by the presence of the PtNp layer.

## Experimental

### Preparation

**Figure 1:**
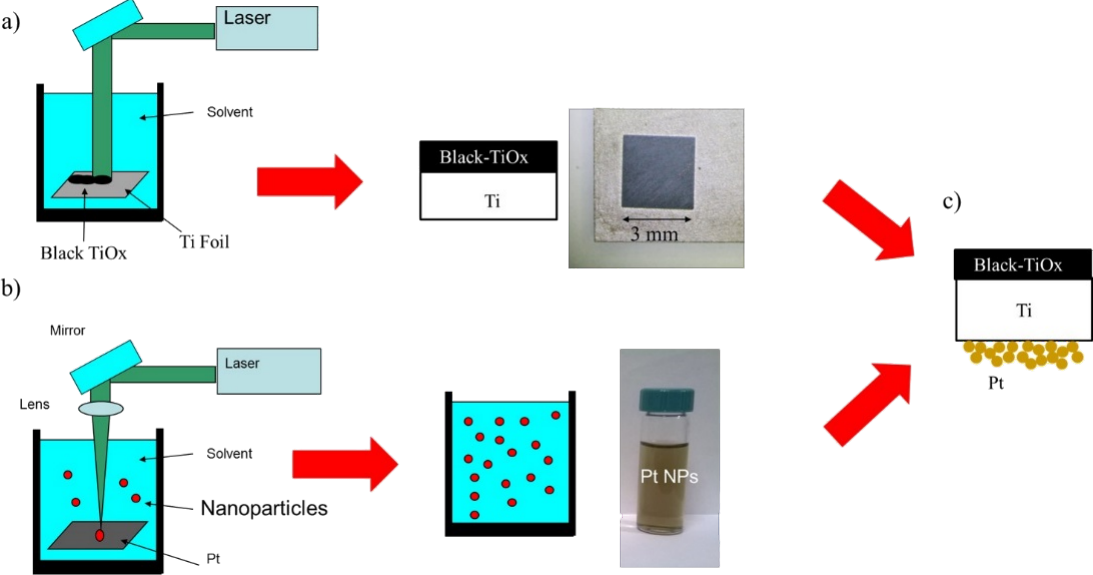
Schematic representation of the sample preparation steps: (a) laser irradiation of the Ti foil in order to form the black TiO*_x_* layer; (b) synthesis of Pt nanoparticles via laser ablation in water; (c) schematic representation of the black TiO*_x_*/Ti/PtNp multilayer structure after the deposition of the Pt nanoparticles on the rear side of the irradiated Ti foil.

The synthesis of the TiO*_x_* film was performed by irradiating a titanium metal foil (Goodfellow, purity 99.9%, as rolled) by a diode-pumped Nd:YVO_4_ laser operating at wavelength of 532 nm, a repetition rate of 20 kHz, and a pulse duration of 15 ns to give a fluence of 2.0 J/cm^2^. The scanning speed was kept at 100 mm/s. The laser was focused using a lens (focal length of 20 cm), on the bottom of a vessel filled with 9 mm of deionized Milli-Q water (resistivity 18 MΩ·cm) above the sample. The sample was irradiated at a fluence of 2 J/cm^2^. A schematic representation of the sample preparation procedure is shown in Figure 1. During laser irradiation, TiO_2_ nanoparticles were obtained in solution, as also reported in a previous publication [24,26], and a thin black layer of TiO*_x_* was formed on the top of the Ti foil (Figure 1a). The samples have a surface area of 0.7 cm^2^.

The synthesis of platinum nanoparticles (PtNps) was performed by pulsed laser ablation in liquid by irradiating a Pt metal foil (Sigma Aldrich, purity 99%) with a Nd:YAG laser (Giant G790-30) at 1064 nm (10 ns pulse duration, 10 Hz repetition rate). The laser was focused using a lens (focal length of 20 cm) on the bottom of a Teflon vessel filled with 5 mL of deionized Milli-Q water (resistivity 18 MΩ·cm). The sample was irradiated at a fluence of 20 J/cm^2^. Pt nanoparticles, dispersed in water, are stable for some months. The synthesis of Pt nanoparticles is represented in Figure 1b.

Samples for photo-degradation tests were realized by depositing some drops (about 2 mL) of the PtNps solution on the rear side of the titanium foil at 90 °C and waiting for evaporation of the water. A continuous layer of platinum nanoparticles adhered to the substrate is formed, leading to the black-TiO*_x_*/Ti/PtNp multilayer structure depicted in Figure 1c.

### Methods

SEM images were acquired by using a field emission SEM (Gemini Zeiss SUPRATM25) at a working distance of 5–6 mm, using an electron beam of 5 keV and SE detector.

The UV–vis spectrum was collected using a Perkin-Elmer Lambda40 spectrometer in the wavelength range 350–900 nm with an integrating sphere (Labsphere 20). The IR spectra were collected using an FTIR spectrometer (Perkin-Elmer Spectrum 1000) in the range 350–7000 cm^−1^. The absorbance was calculated with the formula: *A* = 100 – *R* where *R* is the reflectivity as percentage.

The crystalline structure of the Ti and TiO*_x_*/Ti samples was determined by grazing angle (0.5°) X-ray diffraction by using a Bruker D-9000 instrument (Cu Kα) and Bruker diffraction suite software for the diffraction analysis.

Rutherford backscattering spectrometry (RBS) measurements were run using a 2 MeV He^+^ beam with a scattering angle of 165° in normal incidence. The RUMP software was employed for the analysis of the RBS spectra.

In order to evaluate the photocatalytic activity of the TiO*_x_* nanostructured film, the methylene blue (MB) discoloration test was performed [24]. The apparent photon efficiency in the UV range was calculated according to ISO10678:2010 [27]. The quantum efficiency (QE) was calculated as the ratio between the apparent photon efficiency and the measured absorbance in the wavelength range of the light source. QE represents the ratio between the number of molecules degraded for unit of time (and area) and the number of photons adsorbed by the sample per unit of time (and area). The UV light source (TL 8W BLB 1FM, Philips) spectrum was centred at 368 nm with a FWHM of less than 10 nm, the measured irradiance was about 1.5 mW/cm^2^.

## Results and Discussion

**Figure 2:**
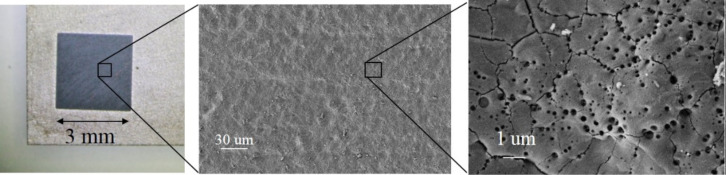
Left: Photograph of the irradiated sample. Middle: low-magnification SEM image of the surface after laser irradiation. Right: High-magnification image of the surface of black TiO*_x_*.

When a high energy, nanosecond laser pulse is focused on a Ti foil, a hot plasma plume forms on the metal surface. The plasma is characterized by high temperature and pressure (≈4700 °C and ≈10^7^ Pa, respectively) [28]. The plume expansion is slowed down by the presence of liquid. As a consequence, inside the plasma, high temperature and pressure are maintained for several μs. Water dissociates and reacts with titanium atoms ejected from the target, realizing H–Ti–O*_x_*. In addition, a cavitation bubble appears, expands, shrinks and collapses in the timescale of hundreds of μs. The collapse is a complex phenomenon that terminates by depositing a layer of titanium oxides on the Ti target [24,28–29]. The redeposited material is black in colour. The effect of the laser irradiation on the titanium foil is reported in Figure 2. In the left side of Figure 2, the irradiated zone is the black square of the sample. The surface morphology, as reported by SEM, reveals that after irradiation, the black surface is uniformly covered by small structures (Figure 2 centre). A high magnification image (Figure 2 right) shows that they consist of cavities and grain boundaries. It is worth noting that the nanostructure of the film appears similar to the case of irradiation under 1064 nm wavelength [22]. However, in the present condition, the holes on the surface of the irradiated sample appear to be concentrated at the grain boundaries generated on the surface by the laser annealing.

**Figure 3:**
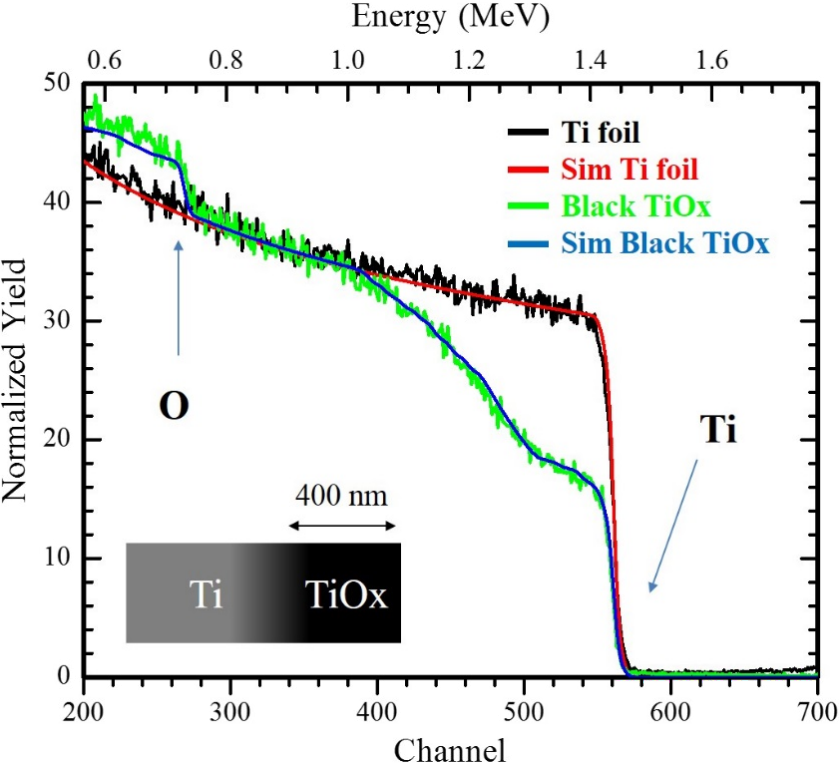
RBS spectra of the TiO*_x_* film and the Ti target. Arrows indicate signals coming from titanium and oxygen, respectively. Simulations of the spectra are also reported. Left lower side: a schematic representation of the irradiated sample is reported.

RBS spectra clearly show the presence of oxygen in the irradiated zone, as indicated in Figure 3. The spectra of the black TiO*_x_* film and the Ti target are reported together with the simulation of their profiles. Close to the surface of the irradiated sample, substoichiometric titanium oxides (such as TiO and Ti_2_O_3_) are revealed by the RUMP simulation [30]. It is observed that the oxygen content decreases to negligible values going from the surface to the inner part of the sample. The RBS profile implies a change in stoichiometry from TiO_2_ on the surface to bulk Ti. The oxidation profile cannot steadily be extracted from the simulation (owing to the complex morphology of the sample). Nevertheless, the average layer thickness can be estimated to be roughly 400 nm.

**Figure 4:**
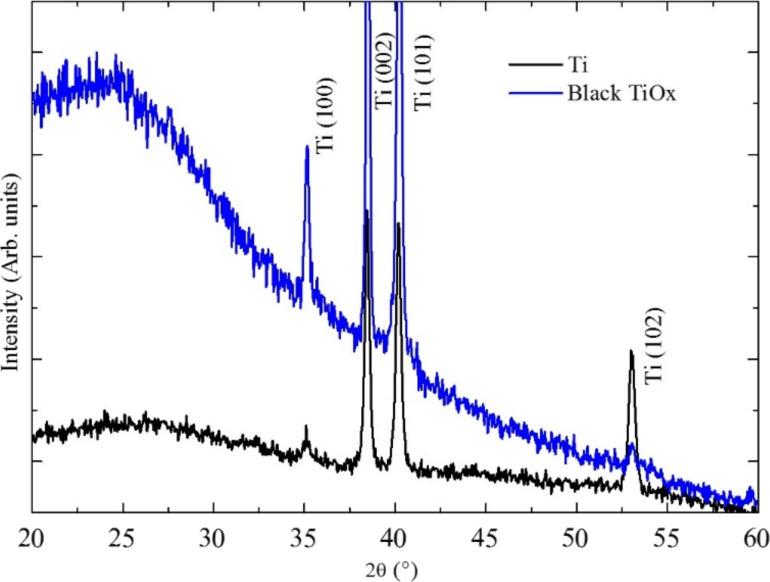
XRD spectra of the TiO*_x_*/Ti film and the Ti foil (before irradiation). The main characteristic peaks of titanium are indicated. The main feature of the black TiO*_x_* sample is the pronounced amorphous signal observed at low scattering angles.

In Figure 4, the XRD spectra of the TiO*_x_* film is shown. In the same figure the spectra of the Ti substrate is shown for comparison. The peaks at about 35°, 38°, 40°, and 53° are apparent in both spectra and they are related to the metallic Ti substrate. The presence of an amorphous phase is clearly recognizable in the spectrum in the low angle range. We ascribe such signal to an amorphous titanium oxide layer.

**Figure 5:**
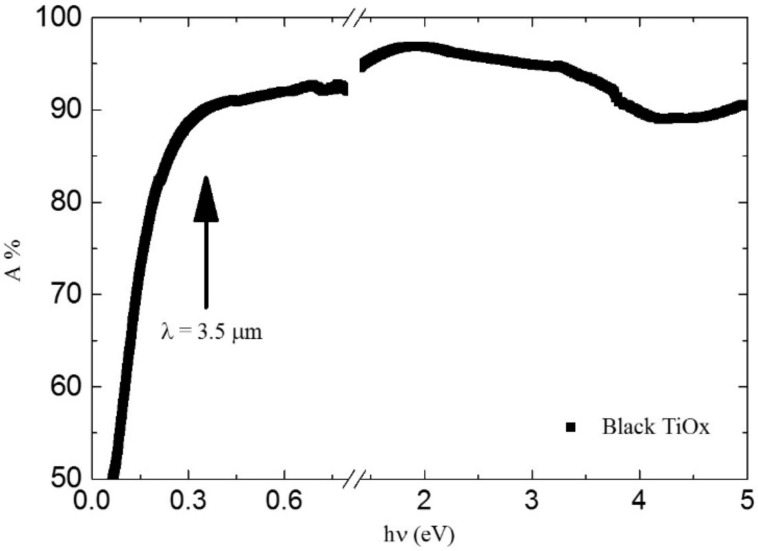
Absorbance spectra of the TiO*_x_*/Ti film in the IR, visible and UV spectral range.

The absorbance spectra (1 − *R* %) of the black TiO*_x_* film is reported in Figure 5. The measured absorbance is higher than 90% in the range from 0.35 to 5 eV (from 3.5 to 0.25 µm in wavelength). The high absorption and black coloration is ascribed to reduced oxidation states of Ti (electrons in the Ti 3d states) and to the presence of H in association with an oxygen vacancy [23]. It is worth noting the obtained black TiO*_x_* has very high absorbance in the full range of solar radiation: in particular, solar radiation spans from 0.5 eV (2.5 µm) to 5 eV (250 nm).

Although it depends on other concomitant surface processes, photocatalytic activity benefits from the high absorption in UV–vis and IR ranges. Indeed, the presence of hydrogen (either interstitial, in TiH or in OH complexes) can effectively enhance the photocatalytic performance of the film [2,6,20–22,24–25]. Furthermore, H-rich zones on the surface can enhance electron scavenging, preventing recombination [2].

**Figure 6:**
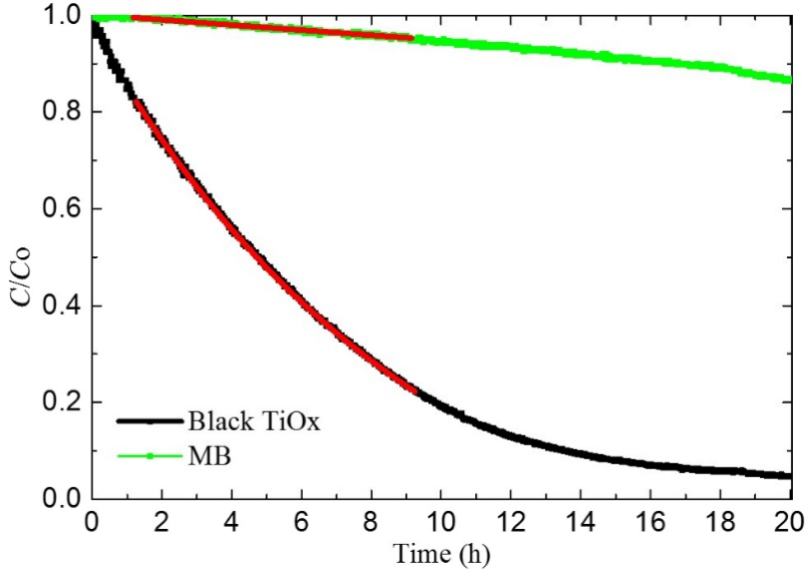
UV discoloration of methylene blue (MB) dye in the presence of TiO*_x_*/Ti/Pt foil. The discoloration of a MB solution without photocatalyst is also reported for comparison purposes.

In the present work, the photocatalytic activity is measured by using the methylene blue (MB) discoloration method [27]. Samples for discolouration measurements were prepared by sandwiching the Ti substrate between a TiO*_x_* layer (irradiated surface) and a Pt nanoparticle layer, thus realizing a TiO*_x_*/Ti/PtNp monolithic chemical diode. The Pt nanoparticles were 20 nm in diameter [29,31] as measured by dynamic light scattering. They were deposited on the rear side of the sample as shown in Figure 1b and discussed in the experimental section. In Figure 6, the concentration of MB under UV irradiation in the presence of black TiO*_x_* material (black) and without the photocatalyst (green) are reported. The MB concentration follows a pseudo first-order kinetic law in the first 10 hours of irradiation. The discoloration rate constant *k* is measured by fitting the curve in Figure 6 with the following formula: *C*/*C*_0_ = e^−^*^kt^*. A discoloration rate *k* of 0.22 ± 0.01 and 0.01 ± 0.01 h^−1^·cm^−2^ was measured for TiO*_x_*/Ti/PtNps and MB, respectively. By using the above result, a quantum efficiency of 0.041% is calculated for the decolouration of methylene blue in water. It is worth noting that the UV quantum efficiency for the TiO*_x_*/Ti/PtNp sample is higher than that reported for standard commercial photocatalytic glass (0.025%) [32].

The photo-activity is mainly due to oxidation of MB molecules adhered to the surface of the TiO*_x_* [12]. This reaction is mainly mediated by holes. In a photocatalytic process driven by titania, electrons have to be transferred (contemporaneously to holes) to the solution in order to maintain charge neutrality in solution. An accumulation of electrons in the material causes an increase of the Fermi level and an increase of the recombination rate of *e*–*h* couples, with a consequent decrease of the photo-activity. Despite some controversial debates in the literature in the past years (mainly regarding the potential activity of the amorphous phase), it has been recently reported that both the presence of an amorphous phase or different crystalline phases (i.e., anatase and rutile, as in the case of the Evonik Aeroxide P25^®^, the former Degussa-P25^®^ powder) can be beneficial in terms of photocatalytic activity [20,24]. In the material presented in this work, the crystalline content is highly reduced (if not absent) with respect our previous work [22]. Regardless, the catalytic performance is remarkable, thus indicating that the role of electron scavenging is the key to prevent recombination and boost the photochemical reactions occurring at the surface. We realized a platinum nanoparticle layer in order to favour the scavenging of electrons due to the high affinity of Pt with oxygen [33] and the high surface/mass ratio of the nanoparticle layer. We observed the presence of a series of substoichiometric oxides (as reported by RBS analysis) that allows for good electrical contact between the black TiO*_x_* surface and the Pt nanoparticle layer on the rear side of the film through the metallic titanium substrate. A higher photo-activity is achieved when holes interact with adhered molecules on the surface by means of surface trap states. In amorphous TiO*_x_*, free holes interact with Ti^+4^ cation surface trap states [12], superficial Ti interstitials [34–35], or hydrogen complexes (realised during the synthesis of the material). These defects on the surface favour the trapping of holes in specific superficial sites and facilitate the interaction with adhered molecules.

## Conclusion

In conclusion, we have proposed a new, simple, scalable and environmentally friendly methodology for synthesizing black TiO*_x_* by using laser irradiation in liquid. We used a commercial, industrial laser with a high repetition rate in order to synthetize black TiO*_x_*. We also noted that this methodology has important advantages with respect to the commonly used synthesis techniques. The obtained material has an extremely high absorbance in the IR, visible and UV spectral range. Although it is composed of amorphous titanium oxide, it is able to degrade pollutants (methylene blue) dissolved in solution at a high rate (QE = 0.041%). This high activity is ascribed to the presence of trap states for holes in the illuminated surface and the a stacked, layered structure (TiO*_x_*/Ti/PtNps) in which the Pt nanoparticle layer (on the rear side of the sample) permits the scavenging of electrons.
